# Cyclooxygenase-2-Mediated Up-Regulation of Mitochondrial Transcription Factor A Mitigates the Radio-Sensitivity of Cancer Cells

**DOI:** 10.3390/ijms20051218

**Published:** 2019-03-11

**Authors:** Fan Tang, Rui Zhang, Jun Wang

**Affiliations:** 1 Key Laboratory of High Magnetic Field and Ion Beam Physical Biology, Chinese Academy of Sciences, No. 350 of Shushanhu Road, Hefei 230031, China; tf268@mail.ustc.edu.cn (F.T.); st1234@mail.ustc.edu.cn (R.Z.); 2 School of Graduate Students, University of Science and Technology of China, Hefei 230026, China

**Keywords:** radio-sensitivity, TFAM, COX-2, mitochondrial fragmentation, p38-MAPK

## Abstract

Mitochondrial transcription factor A (TFAM) regulates mitochondrial biogenesis, and it is a candidate target for sensitizing tumor during therapy. Previous studies identified that increased TFAM expression conferred tumor cells resistance to ionizing radiation. However, the mechanisms on how TFAM are regulated in irradiated tumor cells remain to be explored. In this research, we demonstrated the contribution of cyclooxygenase-2 (COX-2) to enhancing TFAM expression in irradiated tumor cells. Our results showed TFAM was concomitantly up-regulated with COX-2 in irradiated tumor cells. Inhibition of COX-2 by NS-398 blocked radiation-induced expression of TFAM, and prostaglandin E2 (PGE2) treatment stimulated TFAM expression. We next provided evidence that DRP1-mediated mitochondrial fragmentation was a reason for TFAM up-regulation in irradiated cells, by using small interfering RNA (siRNA) and selective inhibitor-targeted DRP1. Furthermore, we proved that p38-MAPK-connected COX-2, and DRP1-mediated TFAM up-regulation. Enhanced phosphorylation of p38 in irradiated tumor cells promoted DRP1 expression, mitochondrial fragmentation, and TFAM expression. NS-398 treatment inhibited radiation-induced p38 phosphorylation, while PGE2 stimulated the activation of p38. The results put forward a mechanism where COX-2 stimulates TFAM expression via p38-mediated DRP1/mitochondrial fragmentation signaling in irradiated tumor cells, which may be of value in understanding how to sensitize cancer cells during radiotherapy.

## 1. Introduction

Mitochondrial transcription factor A (mtTFA, mtTF1, TFAM) is critical in regulating mitochondrial DNA (mtDNA) transcription, packaging and copy number [1,2,3]. It was pointed out that suppressing the expression of TFAM inhibited the proliferation of cancer cells [4,5], increased the sensitivity of cancer cells to chemotherapeutic drugs or ionizing irradiation [6,7,8], and triggered apoptosis [9]. The increased expression of TFAM can be considered as a prognosis marker for poor clinical outcomes of specific type of cancer [10]. At the same time, the decreased expression of TFAM and mitochondrial dysfunction was associated with the pathogenesis of brain and bone diseases [11,12]. TFAM can be up-regulated by ionizing radiation in cancer cell lines, to decrease radiation induced cell death [13]. However, the mechanisms on how TFAM is regulated in irradiated tumor cells remain largely known.

Cyclooxygenase-2 (COX-2), encoded by the *PTGS2* gene, plays important roles in tumorigenesis and inflammation [14,15,16]. The increased expression of COX-2 is considered as a marker for the proliferation of tumor cells [17]. COX-2 plays a critical role in the production of prostaglandin E2 (PGE2). Previous studies showed that COX-2-derived PGE2 induced Id1-dependent radiation resistance and self-renewal in experimental glioblastoma [18]. Other studies have confirmed that the inhibition of COX-2 expression increases the sensitivity of cancer cells to radiation, and COX-2 signaling is a potential therapeutic target for consolidating cancer treatment [19,20,21]. It was reported that a majority of COX-2 in tumor cells were co-localized with heat shock protein-60 in mitochondria, and the mitochondrial localization of COX-2 might confer resistance to apoptosis in different cancer cell lines [22]. Dynamin-related protein 1 (DRP1), a key mediator of mitochondrial fragmentation, is encoded by the *DNM1L* gene [23]. Recent studies have shown that radiation-induced the localization of DRP1 to the mitochondria, and accelerated mitochondrial fragmentation [24]. Preventing mitochondrial fragmentation impaired mitochondrial functions, and led to the loss of mitochondrial DNA [25], indicating that the potential association between mitochondrial morphologies and TFAM was involved in the regulation of mitochondrial biogenesis [3,26,27].

Both TFAM and COX-2 contribute to the resistance of cancer cells to radiation, and they are considered as potential targets for improving the efficacy of radiation treatment in cancers. Besides, they are mitochondrial proteins, and affect mitochondrial functions. Therefore, in this research, we aimed at exploring the interconnections between TFAM and COX-2 in irradiated cancer cells. We identified that COX-2 derived PGE2 enhanced the activation of p38-MAPK, which further stimulated DRP1-mediated up-regulation of TFAM. Our results provided new information on the mechanisms for how COX-2 affects mitochondrial functions, and its implications in increasing the sensitivity of cancer cells to radiation during therapy. The results are described in the following section.

## 2. Results

### 2.1. Concomitant Up-Regulation of TFAM and COX-2 in Irradiated Tumor Cells

**Figure 1:**
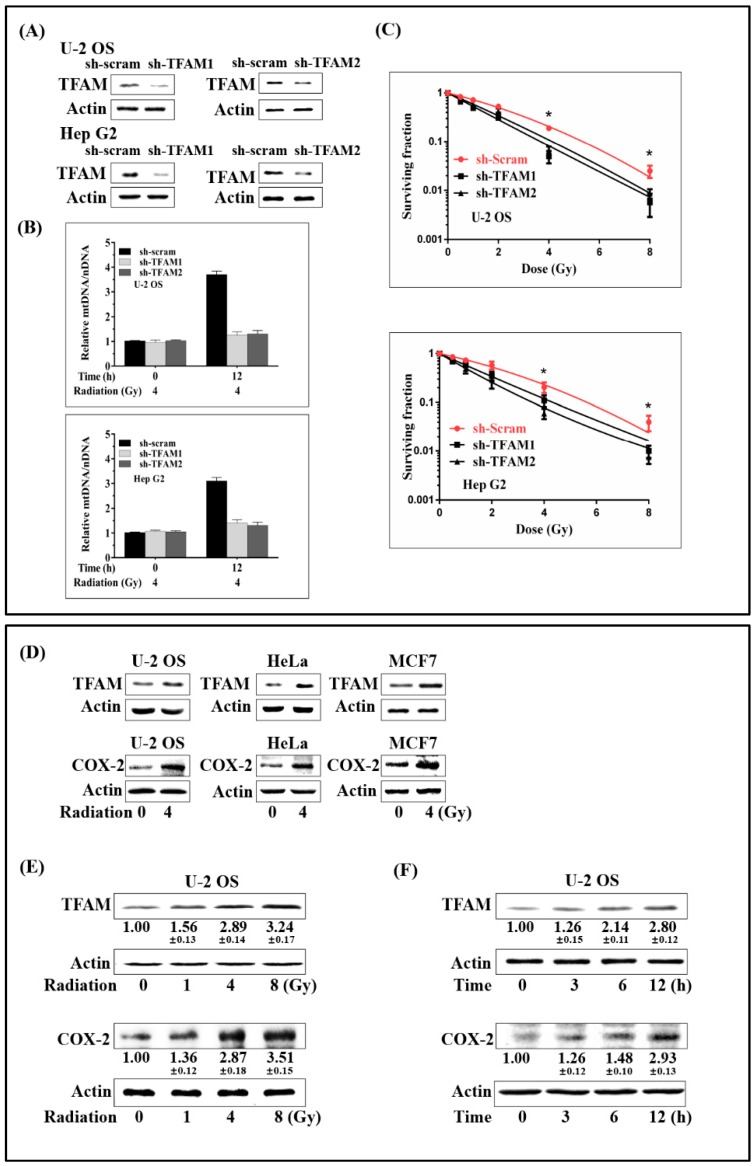
Concomitant up-regulation of TFAM and COX-2 in irradiated tumor cells. (**A**) Construction and verification of *TFAM* knockdown U-2 OS and Hep G2 cell lines. (**B**) Relative Mitochondrial DNA (mtDNA) copy number in irradiated control (sh-scram) and *TFAM* knockdown (sh-TFAM) cells. (**C**) The surviving fraction of the control (sh-scram) and TFAM knockdown (sh-TFAM) U-2 OS and Hep G2 cells. (**D**) Tumor cell lines were irradiated with 4 Gy of γ-rays. 12 h later, TFAM and COX-2 expression was analyzed by immunoblotting. (**E**) U-2 OS cells were irradiated with different doses of γ-ray. After 12 h, the expression levels of TFAM and COX-2 were analyzed by immunoblotting. (**F**) U-2 OS cells were irradiated with 4 Gy of γ-rays. At different time points after radiation, the expression levels of TFAM and COX-2 were analyzed by immunoblotting, respectively. * *p* < 0.05.

TFAM-knockdown U-2 OS and Hep G2 cells were established by transfecting short hairpin RNA (shRNA) plasmids targeting human *TFAM* (Figure 1A). In TFAM knockdown cells, radiation induced elevation of mtDNA copy number was suppressed (Figure 1B). Clonogenic survival assay was applied to test the role of TFAM in sensitizing tumor cells to γ-ray irradiation. As shown in Figure 1C, plots were fitted according to the linear quadratic model, S = exp (−α × *D* − β × *D*^2^), where S is the surviving fraction, *D* is the radiation dose (Gy), and α and β are the fitting parameters. According to the surviving fraction curves, for U-2 OS cells transfected with scramble shRNA plasmid, the 10% survival dose (*D*_10_) and 37% survival dose (*D*_0_) were 5.32 Gy and 2.7 Gy. The *D*_10_ and *D*_0_ for U-2 OS cells transfected with shTFAM1 and shTFAM2 plasmids were 3.64 Gy, 1.54 Gy and 4.18 Gy, 1.85 Gy, respectively. For Hep G2 cells transfected with scramble shRNA plasmid, the 10% survival dose (*D*_10_) and 37% survival dose (*D*_0_) were 5.62 Gy and 2.9 Gy, respectively. The *D*_10_ and *D*_0_ for Hep G2 cells transfected with shTFAM1 and shTFAM2 plasmids were 4.38 Gy, 1.83 Gy, and 3.49 Gy, 1.44 Gy respectively. These results indicated that the knockdown of TFAM expression increased the sensitivity of U-2 OS and Hep G2 cells to radiation. Since COX-2 has been reported to be a pro-survival protein in a wide range of tumor cells, we next detected the expression levels of TFAM and COX-2 after γ-ray irradiation. As shown in Figure 1D, 12 h post-4 Gy γ-ray radiation, the expression levels of both TFAM and COX-2 in U-2 OS, HeLa, and MCF7 cells displayed notable enhancement. We then checked the radiation dose dependency of TFAM and COX-2 expression in U-2 OS cells, 12 h post radiation. As shown in Figure 1E, the expression levels of TFAM and COX-2 were enhanced, with the dose increasing from 1 Gy to 8 Gy, to around 3- and 3.5-fold, compared to those observed in the non-irradiated cells. Next, the time-course for the expression of TFAM and COX-2 were investigated in U-2 OS cells (Figure 1F). The expression levels of TFAM and COX-2 increased within 12 h after radiation, reaching around 2.8- and 3-fold, compared to the non-irradiated controls respectively. Also, due to TFAM up-regulation decreasing the sensitivity of tumor cells to radiation, and because it was concomitantly up-regulated with COX-2, we then aimed at determining whether COX-2 affected the expression of TFAM in irradiated cells.

### 2.2. Activation of COX-2 Up-Regulates TFAM in Irradiated Cells

**Figure 2:**
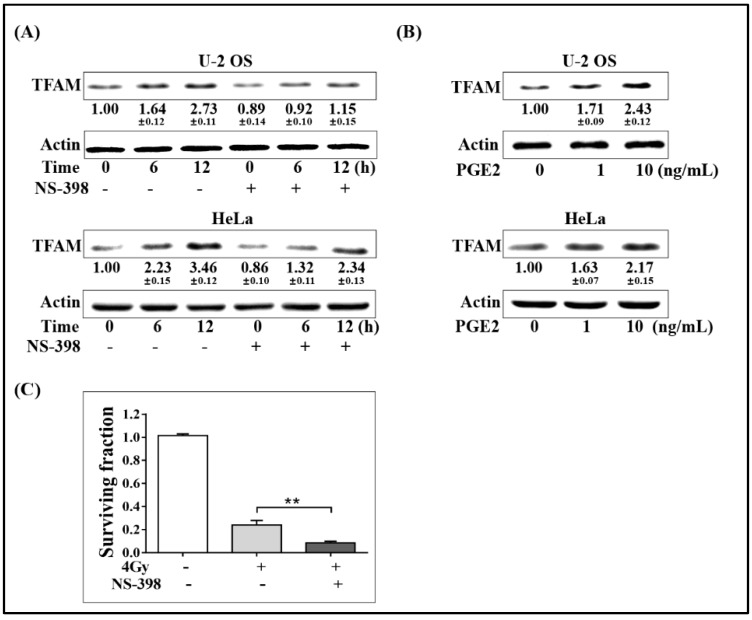
Activation of COX-2-up-regulated TFAM in irradiated cells. (**A**) U-2 OS and HeLa cells were pretreated with NS-398 (20 μmol/L) for 6 h, and then irradiated with 4 Gy γ-ray. The expression levels of TFAM were analyzed by immunoblotting. (**B**) U-2 OS and HeLa cells were incubated with 1 or 10 ng/mL PGE2 for 12 h. The expression levels of TFAM were analyzed by immunoblotting. (**C**) U-2 OS cells pretreated with NS-398 (20 μmol/L) or vehicle for 6 h. Then clonogenic survival assay was performed with radiation dose of 4 Gy. ** *p* < 0.01.

To test whether COX-2 contributed to the up-regulation of TFAM or not, the selective COX-2 chemical inhibitor NS-398 was added into cell culture medium 6 h before 4 Gy γ-radiation at a final concentration of 20 μmol/L. At 6 and 12 h post-radiation, the expression levels of TFAM in U-2 OS and HeLa cells were detected. As displayed in Figure 2A, the addition of NS-398 obviously inhibited the induction of TFAM by radiation. Since NS-398 functions in blocking the enzymatic activity of COX-2, which is desired for the synthesis of prostaglandin, we therefore detected whether prostaglandin E2 (PGE2), the major form of physiological prostaglandin, stimulated the expression of TFAM. As shown in Figure 2B, in U-2 OS and HeLa cells, PGE2 treatment resulted in the elevation of TFAM expression by over 60% at 1 ng/mL, and by over 100% at a final concentration of 10 ng/mL. Further, we tested the effect of NS-398 on the radio-sensitivity of U-2 OS cell under 4 Gy of radiation. As shown in Figure 2C, 4 Gy radiation decreased the cell-surviving fraction to around 24%, compared to that of the non-irradiated cells. NS-398 pre-treatment resulted in a further decrease of the surviving fraction to around 8%. This confirmed the role of COX-2 in stimulating TFAM expression.

### 2.3. Mitochondrial Fragmentation Mediated by DRP1 Up-Regulates TFAM in Irradiated Cells

**Figure 3:**
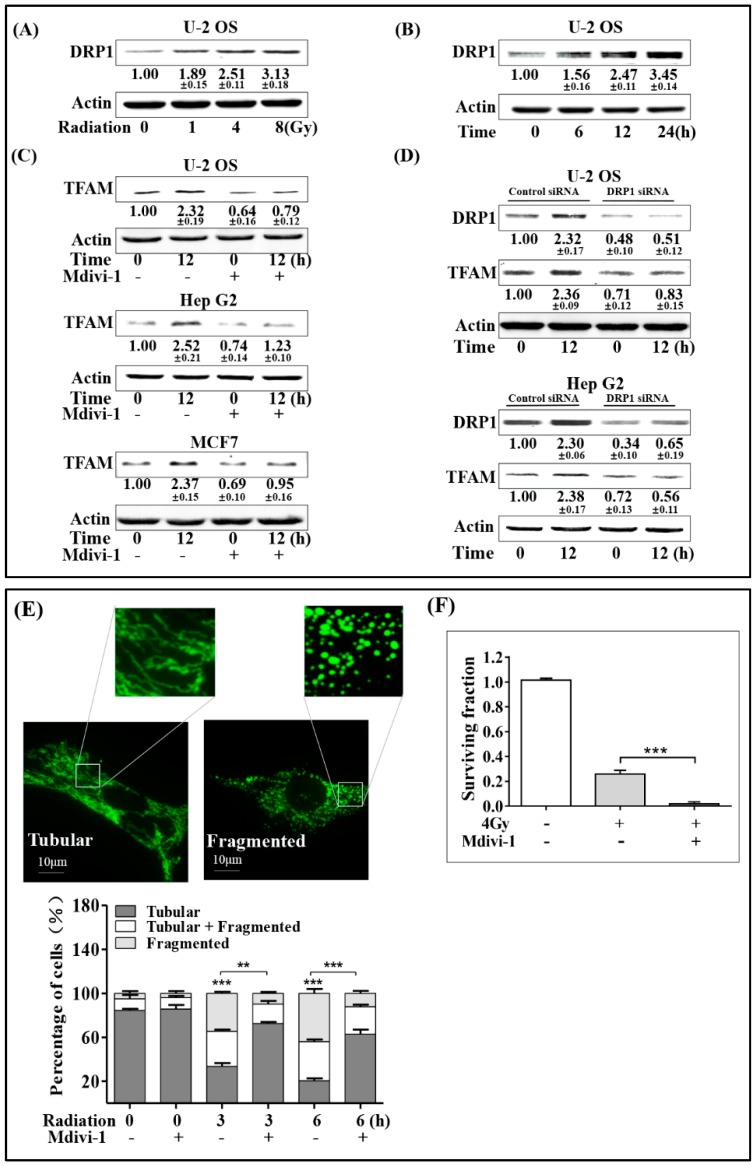
Mitochondrial fragmentation mediated by DRP1 up-regulates TFAM in the irradiated cells. (**A**) U-2 OS cells were irradiated with different doses of γ-rays, and incubated for 12 h. Then, the expression levels of DRP1 were analyzed by immunoblotting. (**B**) Expression levels of DRP1 in U-2 OS cells exposed to 4 Gy γ-rays at the indicated time points post-exposure. (**C**) U-2 OS, Hep G2, and MCF7 cells were pretreated with Mdivi-1 (20 μmol/L) for 1 h, and then irradiated by 4 Gy γ-rays. The expression levels of TFAM were analyzed by immunoblotting. (**D**) U-2 OS and Hep G2 cells were transfected with DRP1 small interfering RNA (siRNA) or control siRNA, and incubated for 48 h. Then, these cells were treated by 4 Gy γ-rays. After a further 12-hr incubation, the expression levels of DRP1 and TFAM were analyzed by immunoblotting. (**E**) Mitochondrial morphologies in vivo were analyzed in mito-GFP transfected U-2 OS cells. Cells were irradiated by 4 Gy γ-rays. (**F**) U-2 OS cells were pre-treated with Mdivi-1 (20 μmol/L) or vehicle. Then clonogenic survival assay was performed with a radiation dose of 4 Gy. Scale bar: 10 μm.** *p* < 0.01 *** *p* < 0.001.

Mitochondrial fragmentation is associated with the biogenesis of mitochondria. DRP1 mediates mitochondrial outer membrane fission in mammalian cells, and it is an important regulator of mitochondrial fragmentation. Given these facts, we next investigated whether DRP1 was involved in radiation-induced TFAM up-regulation. Firstly, the expression of DRP1 in irradiated U-2 OS cells was investigated. As shown in Figure 3A,B, radiation enhanced DRP1 expression in a dose-dependent manner within the range of 0 to 8 Gy. Besides, DRP1 expression was enhanced with post-radiation time elongation within 24 h, reaching a level of around 1.5-folds at the 6 h time point compared, to that in the non-irradiated cells. Then, the *DRP1* small interfering RNA (siRNA) and the selective chemical inhibitor Mdivi-1 were used to check the effect of DRP1 on radiation-induced TFAM expression. A final concentration of 20 μmol/L Mdivi-1 was added into the U-2 OS, Hep G2, and MCF7 culture medium 1 h before 4 Gy radiation. Twelve hours after radiation, total cell lysates were collected for analysis. As in Figure 3C, radiation-induced TFAM expression was largely blocked. Similar results were observed in *DRP1* siRNA-treated Hep G2 and U-2 OS cells (Figure 3D). The indicated radiation-induced DRP1 expression contributed to TFAM up-regulation. As mentioned above, DRP1 is an important regulator of mitochondrial fragmentation, we next investigated the association of mitochondrial morphology with TFAM expression. Plasmid pcDNA3.1 with an insertion of DNA sequence coding mitochondria-localization peptide fused turbo-green fluorescent protein (GFP) was transfected into U-2 OS cells, to visualize mitochondria in living cells under a fluorescence microscope. As shown in Figure 3E, around 85% of cells bore tubular mitochondria, and 5% of the cells bearing fragmented mitochondria if the cells were not irradiated. Three and 6 h after 4 Gy radiation, the cells bearing tubular mitochondria decreased to around 33% and 21%, respectively. However, the mitochondrial fragmentation caused by radiation was inhibited by Mdivi-1 pretreatment. Around 72% and 63% of the irradiated cells 3 or 6 h post-radiation displayed tubular morphology. To confirm the effect of DRP1 on TFAM expression, we tested the effect of Mdivi-1 on the radio-sensitivity of U-2 OS cells under 4 Gy radiation. As shown in Figure 3F, 4 Gy radiation decreased the cell-surviving fraction to around 25%, compared to that of the non-irradiated cells. Also, Mdivi-1 pre-treatment resulted in a further decrease of the surviving fraction to around 1%. Taken together with the results of DRP1 and TFAM expression, DRP1-mediated mitochondria fragmentation was the reason for TFAM induction in the irradiated cells. 

### 2.4. Activation of p38-MAPK Enhances DRP1/TFAM Expression

**Figure 4:**
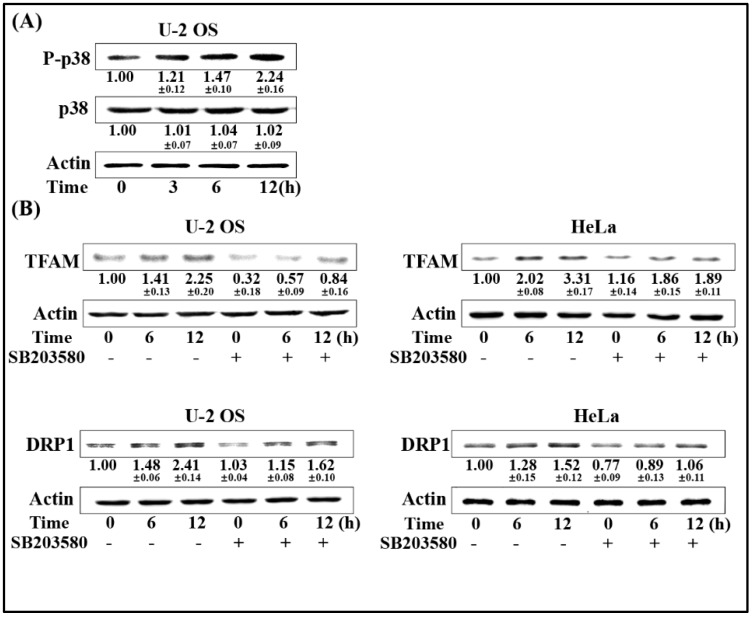
Activation of p38-MAPK enhances DRP1/TFAM expression. (**A**) U-2OS cells were exposed to 4 Gy of γ-rays. At the indicated time points, the irradiated cells were collected and the expression levels of phosphorylated p38 (P-p38) were determined by immunoblotting. (**B**) U-2 OS and HeLa cells were pre-incubated with p38 inhibitor SB203580 (20 μmol/L) for 1 h prior to radiation exposure. Six and 12 h after exposure to 4 Gy radiation, the expression levels of DRP1 and TFAM were analyzed by immunoblotting.

p38-MAPK was reported to be involved in radiation-induced biological responses. To determine the upstream regulator of DRP1/TFAM, the phosphorylation of p38 was firstly estimated. As shown in Figure 4A, the phosphorylation of p38 showed an increasing tendency following 4 Gy radiation within 12 h in U-2 OS cells. We next investigated the association between the activation of p38 and the expression of DRP1/TFAM. Selective p38 inhibitor SB203580 was used to pre-treat U-2 OS and HeLa cells 1 h prior to radiation exposure at a final concentration of 20 μmol/L. Six and 12 h post-4 Gy radiation, the expression levels of DRP1 and TFAM were analyzed by Western blotting. As displayed in Figure 4B, radiation induced expression of both TFAM and DRP1 were attenuated by the addition of SB203580 in the two cell lines, indicating that the activation of p38 by radiation was an upstream regulator of the enhanced expression of DRP1 and TFAM.

### 2.5. COX-2/PGE2 Promotes p38 Phosphorylation and DRP1 Up-Regulation

**Figure 5:**
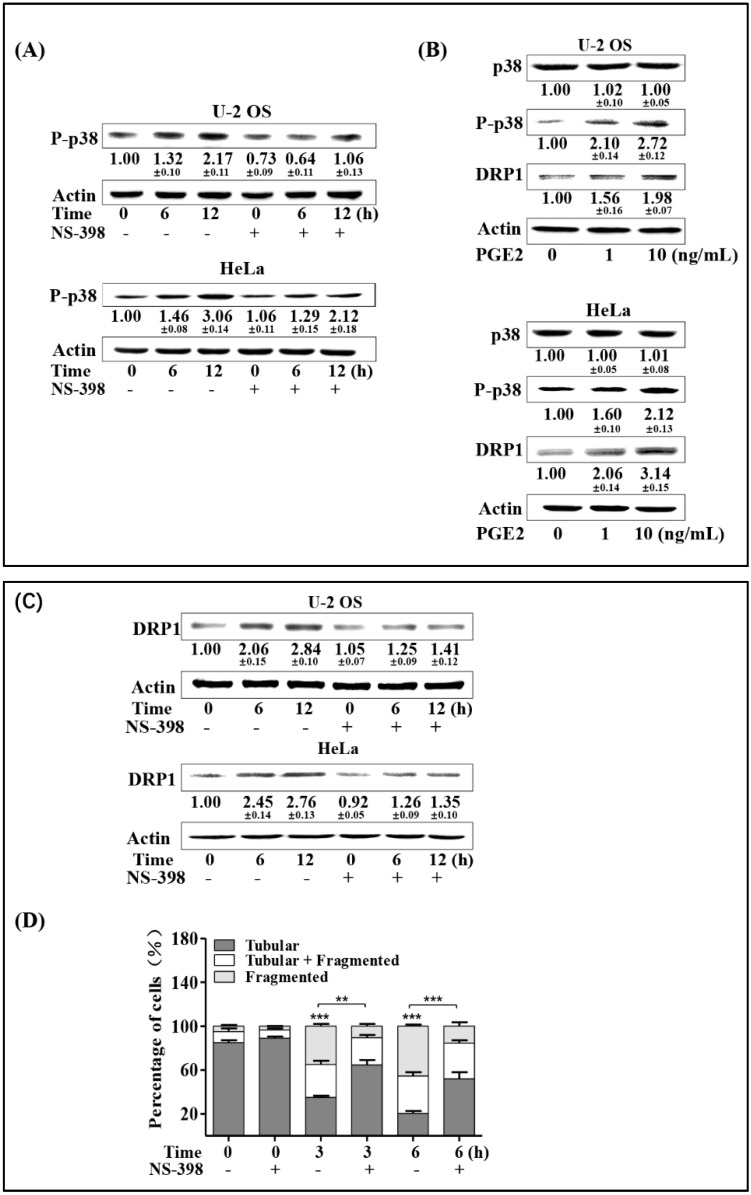
COX-2/PGE2 promotes p38 phosphorylation and DRP1 up-regulation. (**A**) U-2 OS and HeLa cells were treated with COX-2 inhibitor NS-398 (20 μmol/L) for 6 h prior to radiation exposure. Six and 12 h post-4 Gy radiation, expression levels of P-p38 were detected by immunoblotting. (**B**) U-2 OS and HeLa cells were pretreated with 1 ng or 10 ng/mL PGE2. After incubation for 12 h, expression levels of P-p38 and DRP1 were analyzed by immunoblotting. (**C**) U-2 OS and HeLa cells were pre-incubated with COX-2 inhibitor NS-398 (20 μmol/L) for 6 h prior to radiation exposure. At the indicated time points after radiation, cells were collected, and the expression levels of DRP1 were detected by immunoblotting. (**D**) U-2 OS cells were pretreated with NS-398 for 6 h, and then irradiated with 4 Gy of γ-rays. Three and 6 h post-radiation, mitochondrial morphologies were recorded and analyzed. ** *p* < 0.01, *** *p* <0.001.

Since we proved that both COX-2/PGE2 and DRP1 up-regulation mediated by p38 contributed to the enhanced expression of TFAM in irradiated cells, we next investigated whether COX-2/PGE2 affected the phosphorylation of p38 and DRP1 expression. Firstly, we checked the influence of the COX-2 inhibitor NS-398 on radiation-induced phosphorylation of p38. As shown in Figure 5A, NS-398 pre-treated U-2 OS and HeLa cells showed attenuated levels of phosphorylated p38 at 6- and 12-hr time points post-4 Gy radiation, compared to the corresponding groups that were not pre-treated with NS-398, indicating that COX-2 contributed to the activation of p38. This was further confirmed by the results that 1 ng/mL and 10 ng/mL PGE2 could lead to obvious enhancement of p38 phosphorylation (Figure 5B). We next explored whether COX-2/PGE2 also promoted DRP1 expression in U-2 OS and HeLa cells. As shown in Figure 5B,C respectively, DRP1 expression was attenuated by NS-398 pretreatment in cells irradiated by 4 Gy γ-rays, and this was enhanced by PGE2 treatment. To further confirm the effect of COX-2 on DRP1, the mitochondrial fragmentation induced by 4 Gy radiation under the presence of NS-398 was measured with U-2 OS cells bearing plasmid-producing mitochondria-localized turbo-GFP. As shown in Figure 5D, around 85% of the non-irradiated cells bore tubular mitochondria. Three and 6 h after 4 Gy radiation, the cells bearing tubular mitochondria decreased to around 35% and 20%, respectively. However, NS-398 pre-treatment resulted in recovery of the percentage of the irradiated cells displayed tubular mitochondria, to around 64% and 52%. This indicated that COX-2 spurred DRP1-mediated mitochondrial fragmentation. Together with the above results, it was identified that radiation-induced COX-2 facilitated the activation of p38-MAPK, which further enhanced DRP1 expression and its mediated mitochondrial fragmentation.

## 3. Discussion

Previous reports have shown that the expression of TFAM was up-regulated, together with the increase of the mtDNA copy number after α-particle irradiation in human lung adenocarcinoma A549 cells [7]. In the present study, we demonstrated that γ-irradiation up-regulated the expression of TFAM in U-2 OS, Hep G2, HeLa, and MCF7 cells, indicating that the enhanced expression of TFAM is widespread in tumor cells that are exposed to ionizing irradiation. Xie [9] reported that the down-regulation of TFAM inhibited lung cancer cell tumorigenesis, leading to increased apoptotic cell death. Yao [4] determined that microRNA-200a inhibits cell proliferation by targeting TFAM in breast cancer. Han [5] indicated that the over-expression of TFAM enhanced the growth of cancer cell lines, whereas the down-regulation of TFAM inhibited cell growth. Fan [6] found that MiR-199a-3p enhanced breast cancer cell sensitivity to cisplatin by downregulating TFAM. Mei [28] indicated that reduced mtDNA copy numbers caused by TFAM knockdown sensitized the tumor cell lines HEp-2, HNE2, and A549 to chemotherapeutics. Taken with our result that TFAM knockdown sensitized U-2 OS and Hep G2 to ionizing radiation, it could be inferred that the enhanced expression of TFAM was a response of tumor cells to stressed conditions, and that the inhibition of TFAM might be a way for increasing the efficacy of tumor therapy.

COX-2 can be induced by oncogenes, growth factors, and proinflammatory mediators in various types of cells [29]. COX-2 is over-expressed in numerous types of cancer, and it mediates the production of prostaglandins, which stimulate cancer growth and protect the cells against damage by cytotoxic agents [30,31]. Dandekar [32] reported that the COX-2 inhibitor celecoxib augmented chemotherapeutic drug-induced apoptosis by enhancing the activation of caspase-3 and -9 in prostate cancer cells. Liou [22] showed that a majority of COX-2 expression in tumor cells were co-localized with heat shock protein-60 in mitochondria. Another study indicated that mitochondrial-located COX-2 was involved in protecting tumor cells via impairing mitochondrial damage and apoptosis [33]. Chang [34] determined that WNT signaling controlled the radiosensitivity of head and neck cancer cell lines via the COX-2 mediated expression of the DNA repair protein Ku. Besides, further reports showed that the enhanced sensitivity of tumor cells to ionizing radiation under the presence of selective COX-2 inhibitors involved the inhibition of cellular repair from radiation damage and cell cycle redistribution [21]. To our knowledge, there have been no previous reports on whether COX-2 could affect TFAM-mediated biogenesis, to help cells to resist stress conditions. Our results confirmed this hypothesis by providing data to show that selective COX-2 inhibitor NS-398 treatment blocked the induced expression of TFAM in irradiated tumor cells. Prostaglandin E2 (PGE2) is the most abundant prostanoid, and it is produced predominantly from arachidonic acid by tightly regulated cyclooxygenases and prostaglandin E synthases. COX-2-derived PGE2 induced Id1-dependent radiation resistance and self-renewal in experimental mouse glioblastoma [18]. Our results indicated that PGE2 could stimulate TFAM expression in tumor cells without being irradiated. This provided new information on the mechanisms for the regulation of mitochondrial functions by COX-2 under extrinsic stimuli. 

Mitochondria are double-membrane-enveloped organelles. They are highly dynamic, with their normal functions being maintained by continuous morphological changes [35,36]. DRP1 is the master regulator of mitochondrial fragmentation [37]. Parone [25] reported that preventing mitochondrial fragmentation by down-regulating the expression of DRP1 in mammalian cells led to the loss of mitochondrial DNA. Zhang [13] reported that the knockdown of TFAM resulted in the suppression of radiation-induced elevation of mtDNA copy number. In this research, we demonstrated that radiation fragmented mitochondria and up-regulated TFAM. Also, when radiation induced mitochondrial fragmentation was suppressed, TFAM up-regulation was subsequently attenuated, indicating that the morphological changes of mitochondria contributed to radiation-induced TFAM expression. Our work provided results to show that the inhibition of COX-2 by NS-398 decreased the expression of DRP1 and mitochondrial fragmentation in the irradiated tumor cells, which was in line with the findings of Zhou [38], and that the down-regulation of mitochondrial COX-2 inhibited the stemness of nasopharyngeal carcinoma by decreasing the activity of DRP1. Combined with our above results, we identified a signaling mechanism where COX-2 stimulated TFAM expression in irradiated tumor cells via DRP1-mediated mitochondrial fragmentation.

p38 is a member of the MAPKs, and it responds to a variety of stress stimuli, including cytokines, radiation, and heat shock. Its activation in irradiated cells is associated with radiation-induced cell cycle arrest and apoptosis [39,40]. Kim [41] proved that p38 contributes to radiation-induced innate immune responses through inducing the expression of pro-inflammatory cytokines such as TNF-α, IL-6 and IL-12p40. Besides, p38 is involved in the regulation of mitochondrial functions and dynamics. Cryptotanshinone promoted mitochondrial biogenesis in C3H10T1/2 mesenchymal stem cells via AMPK and p38 signaling [42]. Debattisti [43] proved that reactive oxygen species (ROS) activated p38 to affect mitochondrial distribution and motility. It was also reported that p38 chemical inhibitors treatment down-regulated Drp1 in rat brain tissue collected from experimental stroke [44]. In this research, our results showed that p38 was activated in irradiated tumor cells and that its inhibitor repressed radiation-induced up-regulation of DRP1, as well as TFAM. This further confirmed the regulatory role of p38 on mitochondria, and indicates the impacts of mitochondrial morphological changes on TFAM expression and mitochondrial biogenesis. 

Since we proved that COX-2 and p38 positively regulates DRP1/TFAM expression in irradiated tumor cells, we subsequently checked the contribution of COX-2 to p38 phosphorylation. Our results showed that NS-398 treatment decreased radiation-induced p38 phosphorylation, while PGE2 treatment could stimulate it. Kim [45] reported that COX-2 inhibitor SC-236 suppressed nuclear factor-kappa B activation and the phosphorylation of p38 in human mast cell line cells. Zhao [46] found that in HeLa cells overexpressing connexin-26, the knockdown of COX-2 resulted in the attenuated phosphorylation of p38. In addition, Jin [47] determined that PGE2 stimulates the phosphorylation of ERK and p38 to inhibit the ROMK-like small-conductance K (SK) channels, and Ca^2+^-activated big-conductance K channels (BK) in mouse cortical collecting duct cells of. Our results that PGE2 stimulated p38 phosphorylation were consistent with these results, and further confirmed that COX-2 contributes to DRP1/TFAM expression via p38. Besides, Tessner [48] reported that p38 is critical for the enhanced transcription and expression of COX-2 in γ-ray-irradiated human epithelial cells, showing that there was feedback regulation between COX-2 and p38 in irradiated cells.

In conclusion, our current work provides evidence to show that COX-2 contributes to the up-regulation of TFAM, which further helps tumor cells to resist ionizing radiation. We also clarify that this is mediated by COX-2-stimulated p38 activation, and the subsequent enhancement of DRP1 expression and mitochondrial fragmentation. The results of our research put forward a mechanism for the regulation of TFAM in irradiated tumor cells, which may be considered as a candidate sensitization target in cancer radiotherapy.

## 4. Materials and Methods 

### 4.1. Cell Cultures and γ-Irradiation

Human tumor cell lines U-2 OS, Hep G2, HeLa, and MCF7 were from ATCC (Manassas, VA, USA) and cultured in Dulbecco’s modified Eagle’s medium/F12 with 10% fetal bovine serum (Clark Bioscience, Richmond, VA, USA) at 37 °C in a humidified 5% CO_2_ incubator. The γ-ray emitter was a Biobeam GM gamma irradiator (Leipzig, Germany), which contained a ^137^cesium source, with a dose rate of 3.27 Gy/min.

### 4.2. Chemicals and Reagents

The following primary antibodies were utilized: TFAM (1:500; sc-376672; Santa Cruz Biotechnology, Dallas, TX, USA); COX-2 (1:1000; 160112; Cayman Chemical Company, Ann Arbor, MI, USA); P-p38 (1:2000; 612280; BD Biosciences, Franklin Lakes, NJ, USA); DRP1 (1:1000; 12957-1-AP; ProteinTech, Rosemont, IL, USA); β-actin (1:1000; sc-8432; Santa Cruz Biotechnology, Dallas, TX, USA). HRP-linked goat anti mouse or rabbit IgG were purchased from Jackson ImmunoResearch (West Grove, PA, USA). SB203580 was purchased from Selleck Chemicals (Houston, TX, USA). NS-398 was purchased from Abcam (Cambridge, UK) and Mdivi-1 was purchased from Sigma-Aldrich (Merck KGaA, Darmstadt, Germany). PGE2 was purchased from Santa Cruz Biotechnology (Dallas, TX, USA). In this work, SB203580, NS-398, Mdivi-1, and PGE2 were respectively dissolved in DMSO, to prepare 1000-fold stock solutions. Upon usage, equal volumes of vehicle were added into the control groups.

### 4.3. Western Blot Analysis

Cell samples were washed with PBS, and the cell lysate was prepared using RIPA buffer containing a protease inhibitor cocktail (Roche Diagnostics GmbH, Germany) and a protein phosphatase inhibitor cocktail (Sigma, Merck KGaA, Darmstadt, Germany). Protein concentrations were identified by using a BCA kit (Sangon Biotech Co., Ltd., Shanghai, China). The cell lysate (50 μg) was resolved using 10% or 12% SDS-PAGE, and transferred onto polyvinylidene fluoride (PVDF) membrane. Following blocking in 1% skim milk, the PVDF membrane was incubated with the primary antibody at 4 °C overnight, according to the product datasheets. Subsequently, the membrane was washed with PBST (PBS buffer containing 0.1% Tween-20) or TBST (NaCl, Tris-HCL, 0.1% Tween-20) and incubated with the corresponding HRP-conjugated secondary antibody for 2 h at room temperature. Protein bands were visualized using a chemiluminescence substrate (Boster, Wuhan, China) and band density was analyzed with ImageJ software (National Institutes of Health, Bethesda, MD, USA).

### 4.4. Quantitative Real-Time PCR

Cellular DNA was extracted using DNAiso reagent (Takara, Shiga, Japan). The relative mitochondrial DNA (mtDNA) copy number was quantified with a SYBR Green quantitative PCR kit (Takara) using the relative standard curve method. mtDNA content was determined by amplification of 12S ribosomal DNA (rDNA) coded by mtDNA with primers 5′TAACCCAAGTCAATAGAAGCC and 5′CTAGAGGGATATGAAGCACC. Nuclear DNA (nDNA) content was determined by the amplification of the β-actin coding sequence with the primers 5′GAGCGGGAAATCGTGCGTGAC and 5′GGAAGGAAGGCTGGAAGAGTG. The mtDNA/nDNA ratio was used to estimate the relative mtDNA copy number. The PCR conditions were: 95 °C for 30 s, 52 °C for 30 s, and 72 °C for 30 s, 30 cycles.

### 4.5. Clonogenic Survival Assay

Post irradiation, cells were trypsinized, diluted, and seeded into cell culture dishes. After incubation for three weeks, the dishes were washed with pre-warmed PBS, fixed with methanol and acetic acid (*v*/*v* = 9:1), and stained with crystal violet for 15 min. Colonies containing more than 50 cells were counted and plotted.

### 4.6. RNA Interference

Human *TFAM* shRNA (shTFAM) constructs were purchased from OriGene (Rockville, MD, USA). Cells with stable knockdown of TFAM were isolated by transfecting the shTFAM construct and then performing selection under the presence of puromycin. The siRNA oligonucleotide targeting human *DRP1* (GCUACUUUACUCCAACUUAUUTT) was synthesized in GenePharma (Shanghai, China). To knock down DRP1, the cells were grown to 70% confluence in a cell culture dish. Then, the DRP1 siRNA was mixed with Lipofectamine 2000 (Thermo Fishier, Carlsbad, CA, USA) and added into the cell culture. After incubation for 48 h, the cells were subjected to radiation treatment and analysis. 

### 4.7. Mitochondrial Morphology Analysis

To analyze the effects of radiation on the dynamic changes of mitochondrial morphology, the DNA segment coding turbo-GFP with the N-terminal fused to the mitochondria-localization sequence of cytochrome C oxidase subunit IV was inserted into pcDNA3.1 plasmid, according to the reading frame. The recombinant plasmid was then transfected into U-2 OS cells. Cells stably expressing mito-tGFP were isolated for mitochondrial morphology analysis. After radiation or chemical inhibitor treatment, at the desired time points, the mitochondrial morphologies were captured under a fluorescence microscope. Mitochondrial morphologies were divided into three types. ‘‘Tubular’’ means that over 70% of cellular mitochondria showed tubular morphology. ‘‘fragmented’’ meant that over 70% of cellular mitochondria showed fragmented morphology. Others were classified as being ‘‘tubular + fragmented’’. At least 200 cells were analyzed for each sample.

### 4.8. Statistical Analysis

All data were presented as the mean ± standard deviation from at least three independent experiments performed in triplicate. Statistical significance between the two groups was evaluated using Student’s t-test with GraphPad Prism 5 (GraphPad Software, Inc., San Diego, CA, USA). Statistical significance between multiple groups was evaluated using one-way analysis of variance with SPSS 12.0 software (SPSS, Inc., Chicago, IL, USA). *p* < 0.05 was considered to indicate a statistically significant difference.
